# Impact of Narrow Band Imaging in Pre-Operative Assessment of Suspicious Oral Cavity Lesions: A Systematic Review

**DOI:** 10.22038/ijorl.2021.51485.2746

**Published:** 2021-05

**Authors:** Carmelo Saraniti, Giuseppe Greco, Barbara Verro, Norhafiza-Mat Lazim, Enzo Chianetta

**Affiliations:** 1 *ENT Clinic, Department of Biomedicine, Neurosciences and Advanced Diagnostic, University of Palermo, Palermo, Italy.*; 2 *Department of Otorhinolaryngology-Head & Neck Surgery, Universiti Sains Malaysia, Health Campus 16150, Kubang Kerian, Kelantan, Malaysia.*

**Keywords:** Cancer, Erythroplakia, Leukoplakia, Narrow band imaging, Oral

## Abstract

**Introduction:**

Narrow band imaging (NBI) is a powerful tool that allows visualizing the mucosal and submucosal vasculature. Among the available diagnostic techniques, NBI is one of the most valid for early detection of oral squamous cell carcinoma (OSCC).

**Materials and Methods:**

We carried out a bibliographic search in PubMed, Scopus and Web of Science databases using relevant keywords. Articles selected were screened by two independent authors based on inclusion and exclusion criteria. Nine papers were singled out according to the eligibility criteria and included in this review. We investigated the articles for pooled sensitivity, specificity, accuracy, positive predictive value and negative predictive value of pre-operative NBI.

**Results:**

The use of NBI examination in the oral cavity revealed higher specificity, sensitivity, positive and negative predictive values and accuracy compared to white light examination for the diagnosis of oral squamous cell cancer (OSCC). In addition, NBI has proved great utility in detecting malignancy features in oral pre-malignant lesions.

**Conclusions:**

This review shows that NBI is a powerful tool for examining oral suspicious lesions. Most of the articles examined revealed high values of sensitivity, specificity, positive predictive value, negative predictive value and accuracy in detection of oral malignant and pre-malignant lesions. Therefore, the use of NBI is highly recommended for the early detection of oral cancer and potentially malignant disorders. Future studies should seek to affirm the validity of NBI and in particular to standardize NBI classification.

## Introduction

Oral cancers are a vast group of cancer type that manifests in different areas of the oral cavity. Oral squamous cell carcinoma (OSCC) is one of the most common histological types of head and neck cancer and its incidence typically increases with age. 

Tobacco and alcohol are well-known risk factors for the development of this cancer, other risk factors included in the etiology comprehend human papillomavirus (HPV) infection, chewing betel quid or radiation exposure. The early detection of the tumor or precancerous lesions and their radical resection is key for oral cancer prognosis. Indeed, several studies show that failure to obtain both superficial and deep clear margins in OSCC lead to an increase of local recurrence and mortality rate of OSCC patients. In recent years several new non-invasive technologies have been introduced for the diagnosis of oral pathology. Among these, the Narrow–Band Imaging (NBI) has shown excellent results in the identification of superficial mucosal lesions of oral mucosa (1).

NBI was first conceived for the detection of gastrointestinal tumors, subsequently, used in identifying early stage mucosal SCC of head and neck (2-4). 

This device uses two narrow bands of light (blue and green), the former at 400-430 nm and the latter at 525-555 nm, to penetrate superficial mucosa and to enhance the visualization of the mucosa surface and the capillary pattern of the superficial layer, in particular the intraepithelial papillary capillary loops (IPCLs) (5). Different IPCL patterns have been used in the various fields of NBI application. In particular, the Takano’s classification is currently the most used for the oral cavity (6); Takano et al modified Inoue’s classification created for esophageal mucosal lesions. 

Normal mucosa has IPCL type I and is characterized by regular vascular arborization; IPCL type II and III show dilated and elongated vasculature pattern respectively; lastly, irregular morphology or ripped blood vessels are classified as type IV. 

IPCL types I and II are typically present in non-neoplastic lesions while patterns III and particularly pattern IV can be predictive of High-Grade Dysplasia (HGD), carcinoma in situ (CIS) and OSCC (7). Since the use of white light (WL) endoscopy only may not to be enough to detect specific neo angiogenic patterns suggestive of pre-malignant and malignant transformation, NBI represent an important tool for the clinician to identify the pre-malignant and malignant mucosal lesion. Moreover, the use of this device, especially in recent years, has been extended to the intraoperative margin assessment in oral cancer surgery and for follow-up care of patients previously treated for OSCC (8). 

Thus, this systematic review aims to evaluate the validity of NBI in terms of sensibility, specificity, positive and negative predictive values (PPV and NPV) and accuracy compared to white light endoscopy for detection and follow up of oral cavity lesions.

## Materials and Methods

Study Criteria

Our literature is based on the review of studies that investigated and evaluated the effectiveness of NBI for aiding the detection of malignant and pre-malignant lesions of the oral cavity. The studies considered had to meet inclusion and exclusion criteria. Inclusion criteria were: original articles reporting the use of NBI in patients affected by oral cavity lesions; studies evaluating at least two of these values between sensitivity (SE), specificity (SP), positive predictive value (PPV), negative predictive value (NPV), accuracy (Acc) for NBI or both WL and NBI; studies published in English in peer-reviewed journals. Studies were excluded if: they were review articles or editorials, opinions, conference abstract or case report; studies that investigated the parameters (SE, SP, PPV, NPV, Acc) for oral together with oropharyngeal lesions.

Search strategy 

Using PRISMA guidelines, research through online databases included PubMed, Scopus and Web of Science (up to 1st April 2020).

A detailed search strategy was performed for each database using relevant keywords, such as “Narrow band imaging” or “NBI”, “oral” or “suspicious oral lesion”, “oral cancer”, “oral cavity”, “oral erythroplakia” or “oral leukoplakia” (Table 1).

**Table 1:** Keywords search strategy

**Database**	**Keywords**		
PubMed Scopus Web of Science	Narrow band imaging OR NBI	AND	Oral Oral cavity Oral cancer Oral lesion Suspicious oral lesion Oral leukoplakia Oral erytroplakia

Selection of studies

**Fig 1:**
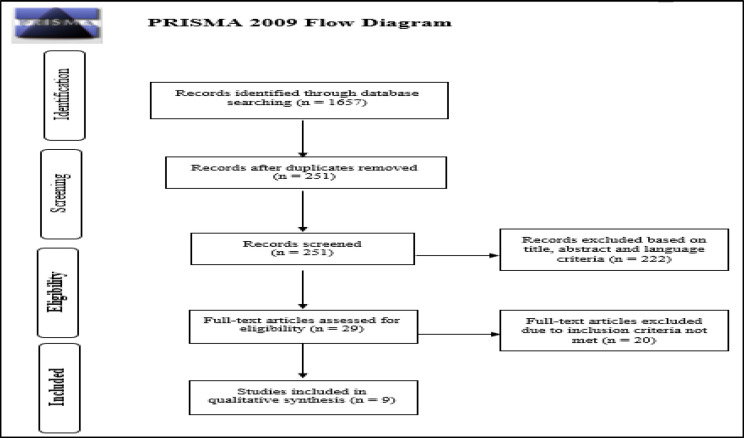
PRISMA Flow Diagram

After removing duplicates, two authors (GG and EC) independently screened titles and abstracts of all studies retrieved from the initial database research. Potentially relevant articles were identified, and their full text obtained for detailed assessment of eligibility according to our inclusion criteria (Figure 1). A third senior author (CS) gave his opinion independently following the same selection criteria when the other two reviewers disagreed. 

***Data extraction***

Details of the selected studies were extracted including authors name, publication year, study type, inclusion and exclusion criteria, number of patients enrolled, number and type of lesions analyzed and in particular the values of sensitivity, specificity, PPV, NPV and accuracy of NBI and eventually WL too (using tissue histopathology as the gold standard comparison) (Tables 2 and 3).

**Table 2:** Statistical values of included studies

**Authors**	**Se (%)**	**Sp (%)**	**PPV (%)**	**NPV (%)**	**Acc (%)**
Vu AN et al (2015)	WL 40.54 (24.77-57.90) NBI 43.24 (34.84-92.97)	WL 78.57 (65.56-88.40) NBI 75.00 (16.35-61.62)	WL 55.56 (35.34-74.50) NBI 53.33 (16.35-61.62)	WL 66.67 (53.99-77.79) NBI 66.67(53.66-78.04)	WL 63.44 NBI 62.37
Upadhyay et al (2019)	WL 75.75 NBI 93.93	WL 76.43 NBI 80	/	/	/
Yang SW et al (2013)	WL 96.30 NBI 87.04	WL 60.08 NBI 93.54	WL 33.12 NBI 73.44	WL 98.75 NBI 97.23	WL 66.25 NBI 92.43
Yang SW et al (2014)	NBI 93.75	NBI 91.49	NBI 78.95	NBI 97.73	NBI 92.06
Shibahara et al (2014)	NBI 92.3	NBI 88.2	/	/	/
Ottaviani G et al (2016)	NBI 64 (41-86) (D1) NBI 99 (96-100) (D2) NBI 72 (49-86) (D3) NBI 98 (93-100) (K)	NBI 96 (88-100) (D1) NBI 89 (80-96) (D2) NBI 93 (86-97) (D3) NBI 100 (100-100) (K)	NBI 78 (56-99) (D1) NBI 61 (39-82) (D2) NBI 59 (32-78) (D3) NBI 96 (91-100) (K)	NBI 92 (86-100) (D1) NBI 98 (93-100) (D2) NBI 98 (90-100) (D3) NBI 100 (100-100) (K)	NBI 93 (86-100) (D1) NBI 89 (79-97) (D2) NBI 95 (90-100) (D3) NBI 99 (97-100) (K)
Yang SW et al (2012)	Criteria I: 15.39 Criteria II: 76.92 Criteria III: 84.62	Criteria I: 60.46 Criteria II: 55.01 Criteria III: 94.56	Criteria I: 6.76 Criteria II: 24.15 Criteria III: 74.32	Criteria I: 79.32 Criteria II: 92.75 Criteria III: 97.06	Criteria I: 53.38 Criteria II: 58.45 Criteria III: 93.0
Yang SW et al (2015)	Criteria III: 80.95	Criteria III: 78.43	Criteria III: 60.71	Criteria III: 90.91	Criteria III: 79.17
Sekine R et al (2015)	NBI 89	NBI 93	/	/	/

Se: sensitivity; Sp: specificity; PPV: positive predictive value; NPV: negative predictive value; Acc: accuracy

**Table 3:** Overall characteristics of included studies

**Authors**	**Type of studies**	**N° of patients**	**N° of lesions**	**Type of lesions**	**Classification**
Vu AN et al (2015)	Prospective Retrospective	95	272	Oral mucosal lesions	Takano’s IPCL classification (with addition of type 0 for IPCL not visible)
Upadhyay et al (2019)	Prospective	38	38	Oral mucosal lesions	IPCL Classification
Yang SW et al (2013)	Retrospective	317	317	Oral Leukoplakia	IPCL Classification
Yang SW et al (2014)	Retrospective	63	63	Oral chronic non-healing ulcers	IPCL Classification
Shibahara et al (2014)	Unclear	121	121	Oral mucosal lesions	Inoue Classification
Ottaviani G et al (2016)	Prospective	114	91	Oral mucosal lesions	IPCL classification in according to Takano description. 0: nonpathological D1: mild dysplastic D2: moderate dysplastic D3: severe dysplastic K: neoplastic
Yang SW et al (2012)	Retrospective	414	414	Oral Leukoplakia	IPCL classification Criteria I: brownish spots and demarcation line with irregular microvasculature patterns Criteria II: well demarcated brownish area with thick dark spots and/or winding vessels Criteria III: IPCL type III and IV
Yang SW et al (2015)	Retrospective	72	72	Oral erythroplakia	IPCL classification Criteria I: brownish spots and demarcation line with irregular microvasculature patterns Criteria II: well demarcated brownish area with thick dark spots and/or winding vessels Criteria III: IPCL type III and IV
Sekine R et al (2015)	Prospective	119	119	Oral mucosal lesions	Takano's classification

## Results

We searched from databases including PubMed, Scopus and Web of Science selecting a total of 1657 articles. After eliminating duplicates (1406), we screened 251 articles for title, abstract and language criteria, and 222 of these were eliminated because not relevant. Out of these, 29 full papers were obtained for screening against the inclusion criteria. A total of 9 studies was eligible to be included in the review.

Preoperative use of NBI and suspicious malignant lesions

Yang et al (9) retrospectively reviewed 63 patients with chronic oral non-healing ulcers. All patients underwent NBI examination and each lesion was classified using IPCL classification. The clinical sensitivity, specificity, PPV, NPV and accuracy for the detection of OSCC by NBI were 93.75%, 91.49%, 78.95%, 97.73%, 92.06% respectively. Most of the IPCL type patterns III and IV were associated with pre-malignant and malignant lesions, with only 4 cases out of 19, among those classified as pattern IPCL III and IV, diagnosed as benign ulcers. Shibahara et al (10) performed NBI endoscopy on 121 patients affected by a wide range of oral pathologies. According to the results, NBI has showed high sensitivity (92.3%) and specificity (88.2%) in early detection of OSCC. In their article, Ottaviani et al (11) presented 91 oral lesions that had been biopsied and classified for different histopathological grades. The authors reported 98% sensitivity, 100% specificity, 96% PPV, 100% NPV and 99% accuracy for neoplasia (indicated as grade “K”). Another article (12) reported 119 oral mucosal lesions assessed with NBI preoperatively. In particular, Sekine pointed out that most of the cases of OSCC were classified as type III or IV, showing sensitivity and specificity of 89% and 93% respectively.

Comparison between WL and NBI

Three of the selected articles compared the use of NBI as opposed to WL for the assessment of oral mucosal lesion in the analysis of their results (13-15). In particular, in Upadhyay et al. study (13), 38 suspicious oral lesions were analyzed first with conventional white-light endoscopy and later with NBI before undergoing a punch biopsy for histopathological diagnosis. In these patients, the efficacy of NBI was higher than WL in terms of sensitivity and specificity. In contrast with these results, the effectiveness of NBI has been questioned in Vu et al article (14). This study reported sensitivity, specificity, PPV, NPV and accuracy of 40.54%, 78.57%, 55.56%, 66.67% and 63.44% respectively for WL, and 43.24%, 75%, 53.33%, 66.67% and 62.37% respectively for NBI. Yang et al (15) carried out a similar study, analyzing 317 patients with oral leukoplakia. In this study, NBI showed a sensitivity of 87.04%, specificity of 93.54%, PPV of 73.44%, NPV of 97.23% and accuracy of 92.43% in predicting high-grade dysplasia and carcinomatous lesions in oral leukoplakia. Also, the study revealed that the combined use of WL and NBI showed a high level of sensitivity (100%) and negative predictive value (100%) in detecting HGD/Tis/Ca lesion in oral leukoplakia. 

NBI and erythro- leukoplakia

In addition to the study concerning NBI and leukoplakia mentioned above (15), Yang et al carried out other two studies (16,17) in which they verified the efficacy of NBI in detecting HGD/Tis/Ca in oral leukoplakia and erythroplakia. Vascular morphology of each lesion was grouped in 3 different criteria in both studies; criteria III corresponded type III and IV IPCL pattern. Sensitivity, specificity, PPV, NPV and accuracy have been calculated in each study and the detection rate of HGD/Tis/Ca in oral leuko- and erythroplakia was significantly higher in cases of NBI criteria III, especially in the 2012 study (16). 

## Discussion

As widely known, early detection of malignant and potentially malignant oral mucosal lesions plays a key role in improving patient prognosis. WL endoscopy may not be enough to detect specific neo angiogenic patterns suggestive of pre-malignant and malignant transformation. This review examined the diagnostic accuracy of NBI in comparison with traditional WL technique or as an independent test for oral malignancy. The papers included in this review tested NBI validity in different clinical contexts. The study about chronic oral non-healing ulcers - lesions poorly responding to medical treatment for at least 3 weeks - was performed by Yang et al (9) in 2014. The study retrospectively analyzed 63 patients, all of them undergoing oral cavity examination with NBI and histopathological evaluation. Among the non-suspicious lesions classified as IPCL type 0, I and II, 97.7 % of them were benign. Nineteen (19) suspicious lesions appeared IPCL type III and IV, with 15 of them were diagnosed as carcinoma (78.9%). The clinical sensitivity, specificity, PPV, NPV and accuracy for the detection of OSCC by NBI were 93.75%, 91.49%,78.95%, 97.73%, 92.06% respectively; the retrospective nature and the possibility of sampling error were the main limitations of the study. Indeed, only highly suspicious oral lesions underwent totally excision while the remaining were subjected to simple biopsies. The interesting issue of the efficacy of NBI in leuko- and erythroplakia lesion was treated by Yang in three papers. In Yang et al.’s 2015 paper, 72 patients with oral homogeneous EP were enrolled (17). All of them underwent endoscopic WL and NBI oral examination. The authors grouped the morphology of the microvasculature patterns of oral erythroplakia based on three different criteria. In particular, Criteria III clustered the IPCL type III and type IV patterns showing a higher detection rate of HGD/Tis/CA compared to the other two criteria. Among the analyzed values stands out a NPV of 90.91%. According to the authors, this high value can support the identification of candidates for the intervention, avoiding the biopsy which could result problematic for some patients. Limitations of the study are the retrospective design and the small number of histological samples. The other two of Yang’s selected papers (15,16) investigate the efficacy of NBI for the detection of high-grade dysplasia and carcinoma in oral leukoplakia. In the first one (16), researchers performed a retrospective case-control study of 414 patients who had been subjected NBI examination and subsequently to biopsy or surgery for oral leukoplakia. The authors established the same NBI Criteria I to III used in the aforementioned study, and a very high NPV for detecting pathological HGD/Tis/CA was found in Criteria III (97.06%). In the second study (15), the authors included 317 patients who received WL and NBI examination and then a biopsy or laser surgery for oral LP. They retrospectively analyzed records of all patients for detecting high-grade dysplasia and carcinomatous lesions in oral LP based on morphological appearances on WL, and microvasculature patterns on NBI. Consequently, sensitivity, specificity, positive and negative predictive values, and accuracy of WL, NBI and WL-NBI jointed use were calculated. According to the NBI classification, the sensitivity, specificity, positive predictive value, negative predictive value, accuracy, amounted to 87.04%, 93.54%, 73.44%, 97.23%, 92.43%, 6.46, and 12.96%, respectively. NBI classification appeared significantly better than the traditional WL classification based on the morphological appearance. This confirmed the validity of NBI as a non-invasive method to identify the malignancy features in oral leukoplakia lesions. As for the study mentioned before, the retrospective nature of the study and the incomplete excision of some lesions represent the study’s limitations. According to the authors, other prospective studies are necessary to validate the role of NBI in the assessment of malignancy in oral leukoplakia. Shibahara et al (10) evaluated specificity and sensitivity of NBI in early detection of OSCC from various oral mucosal lesions. They recruited 121 patients studying the type of vascular loop according to Inoue classification (18) detecting 52 OSCC, 25 leukoplakia, 18 lichen planus, 8 aptha, 6 erythroplakia, 6 dysplasia and 6 others. The observed values were Se 92.3% and Sp 88.2%. The study’s weaknesses are an unclear study design and the lack of some statistical data. Moreover, this is the only study based on Inoue classification. Upadhyay et al in 2019 (13) performed a prospective study analyzing 38 patients with different suspicious oral lesions with NBI endoscopy, dividing them into high and low-risk groups according to IPCL classification. All of them underwent biopsy or excision and histopathology exam. They obtained high values of Se (93.93%) and Sp (80%), and NBI has shown to be a very useful method during the preoperative diagnosis of patients with suspicious oral lesions. Unlike the other studies, Vu et al showed in their article (14) that they found low values of Se (43.24%), Sp (78.57%), PPV (55.56%) and NPV (66.67) for NBI when using histopathology as the gold standard. The cause of this is unclear but the authors suppose that it could be attributed to the smaller sample size, the majority of biopsies taken without input from NBI and the lack of multisite biopsies and examination by a single pathologist. Furthermore, the study showed no strong association between IPCL pattern type and histopathology diagnosis for oral potentially malignant disorders (OPMDs). Ottaviani et al (11) performed in 2016 an interesting prospective study, divided into a preclinical study and a clinical study. In the preclinical study, they evaluated the diagnostic accuracy of NBI in detecting oral cancer induced by an oral carcinogen in mice. The clinical study evaluated the clinical accuracy of NBI in 91 patients. We considered only data from the clinical study on humans. The authors performed a biopsy on all the oral lesions and hired three independent pathologists to analyze the lesions, classifying these in: hyperplastic or inflammatory (0); mild dysplastic (D1), moderate dysplastic (D2); severe dysplastic (D3); neoplastic (K). Two experts in oral medicine and NBI analyzed the lesions’ images acquired before the biopsy by WL and then by NBI lights and tried to attribute a diagnosis selecting between: healthy/non-pathological (0), mild dysplastic (D1), moderate dysplastic (D2), severe dysplastic (D3) and neoplastic (K). Each diagnosis was compared with the histological diagnosis. The statistical analysis of NBI validity was carried out for each histopathologic stage. For example, the results of sensitivity were good for any stage and excellent for D2 (moderate dysplasia, 99%) and K (OSCC, 98%); with 99% accuracy for K. The authors concluded that the NBI is a useful tool in the diagnosis of oral malignant lesions, in the surveillance of pre-malignant oral injuries and during follow-up care of patients with previous diagnoses of OSCC. The experts of NBI images were not blinded from WL assessment. Sekine et al (12) analyzed oral lesions of 119 patients with NBI, reporting Se 89% and Sp 93%. The authors believe that low sensitivity could be due to the difficult analysis of IPCL in mucosal inflammatory lesions. Moreover, they carried out a 3D reconstruction of capillary morphology of the specimens and compared it to IPCL by NBI examination, finding a high match between that capillary patterns, thus proving the effectiveness of NBI in IPCL observation. Our review shows how NBI increases the detection of HGD/Tis/Ca when compared with WL examination. As noted by many authors, the proper use of NBI endoscopy requires a certain amount of experience. An untrained clinician may not recognize essential details to identify risk pattern in the suspicious oral lesions. In our opinion, other medical figures, such as dentists, can use NBI after adequate training. As for any new operator-dependent examination/exam, NBI requires time for the correct interpretation of images. Indeed, the percentage of false positives results high in the first six months of training to then drop down and stabilize at much lower values at a later stage (19). To date, for its features of safety, rapidity and tolerability, NBI could represent a viable replacement for biopsy. Notably, Yang affirms that (17), due to the high NPV, NBI may be considered a valuable diagnostic method in patients unsuitable for biopsy to obtain important information about erythroplakia before surgical intervention and to reduce considerably morbidity, financial costs and time of the physical biopsy. However, extensive prospective studies are necessary. In literature, in addition to the preoperative use, the NBI seems to be a useful tool during the intraoperative management of patients with oral SCC. NBI showed a better assessment of the superficial extension of tumor that resulted in a fewer number of histopathologic positive margins (20,21). 

We have encountered some limitations in this study. First of all, a large percentage of the selected studies has a retrospective design. Secondly, NBI examinations often were performed consecutively after conventional oral examinations (COE) and WL examinations so that NBI images were not blinded by previous WL evaluations. Thirdly, the results could have been affected by the endoscopist's lack of experience which, as seen, could increase the rate of false positive. Lastly, the study can present a chance of sampling error considering that not all lesions were subjected to a total excision but only to simple biopsy.

## Conclusion

The early diagnosis of malignant and pre-malignant lesions in the oral cavity is crucial to provide a better prognosis for patients. The purpose of this review was to evaluate the efficacy of NBI in the detection of oral squamous cell cancer. Based on current evidence, the study found that the use of NBI is strongly recommended for the early detection of oral cancer and potentially malignant disorders. 

In particular, this technique allows better visualization of malignancy features compared to only white light examination, especially in cases of precancerous lesions like erythroplakia and leukoplakia. However, further studies, especially about IPCL classification criteria, are necessary to make sure that future NBI examination could have a direct impact on patient pre-operative management. Indeed, we noticed that different authors often describe the same morphological features of IPCL using different nomenclatures. Therefore, it would be essential to refer to a single and common IPCL classification among the medical community. 

Although several studies show the great value of NBI in the diagnostic phase, there is still little evidence about its intraoperative role. That is the reason why the future challenge will be to consolidate this technique as a valid instrument able to guarantee a complete resection of oral malignant and pre-malignant lesions.
